# COVID-19 in Saudi Patients With Sickle Cell Disease: A Retrospective Multi-Center Study

**DOI:** 10.7759/cureus.17238

**Published:** 2021-08-16

**Authors:** Ohoud Kashari, Badriah Alghamdi, Abdulqader Al-Hebshi, Aljawharah Asiri, Ebtehal Fallatah, Fayez Alshehri, Salihah Alsamiri, Hassan Masmali, Mohammad Nabulsi, Mona Assiri, Turki A Alwasaidi

**Affiliations:** 1 Department of Pediatrics, East Jeddah General Hospital, Jeddah, SAU; 2 Division of Hematology, Department of Pediatrics, Prince Mohammed bin AbdulAziz Hospital, Ministry of National Guard - Health Affairs, Medina, SAU; 3 Department of Pediatrics, King Abdullah International Medical Research Center (KAIMRC), Riyadh, SAU; 4 Faculty of Medicine, Department of Pediatrics, King Saud Bin Abdulaziz University for Health Sciences (KSAU-HS), Riyadh, SAU; 5 Department of Pediatrics, Maternity and Children Hospital, Khamis Mushayt, SAU; 6 Medicine, Royal College of Surgeons, University of Medicine and Health Sciences, Dublin, IRL; 7 Department of Pediatrics, Maternity and Children Hospital, Mecca, SAU; 8 Department of Medicine, King Abdullah Medical Complex, Jeddah, SAU; 9 Department of Pediatrics, Maternity and Children Hospital, Abha, SAU; 10 Division of Hematology, Department of Medicine, Prince Mohammed bin Abdulaziz Hospital, Ministry of National Guard - Health Affairs, Madinah, SAU; 11 Department of Medicine, King Abdullah International Medical Research Centers (KAIMRC), Medina, SAU; 12 Department of Medicine, College of Medicine, Taibah University, Medinah, SAU; 13 Faculty of Medicine, Department of Pediatrics, King Saud Bin Abdulaziz University for Health Sciences (KSAU-HS), Jeddah, SAU

**Keywords:** sickle cell disease, covid-19, saudi arabia, management, prognosis

## Abstract

Background

The prevalence of sickle cell disease (SCD) within Saudi Arabia is relatively high, with an estimated 145/10,000 cases. There is an urgent need for researching many aspects of the Coronavirus disease of 2019 (COVID-19) due to the widespread of the virus among SCD patients in Saudi Arabia. The aim of this study is to determine how COVID-19 affects SCD patients in order to reach the best strategy for their management protocols.

Methods

This is a retrospective chart review study from a multi-center in Saudi Arabia that evaluated a total of 33 patients with sickle cell anemia/disease who were confirmed to have COVID-19. The diagnosis of COVID-19 was confirmed by using the reverse transcription-polymerase chain reaction (RT-PCR) tests based on the nasopharyngeal swabs of the included patients.

Results

The mean age of patients was 10.75+9.11 years, and nearly all patients (n= 32; 96.9%) were Saudi, and 48.4% of them were females. Twenty-two patients were admitted (59.5%); the main reasons for admission included vaso-occlusive crisis (VOC) only (n= 6; 27.3%), fever (n= 6; 27.3%), acute chest syndrome (n= 5; 22.7%), and VOC combined with other conditions (n= 4; 18.2%). During hospitalization, 54.1% of the patients received at least one medication, while antibiotics (54.1%), analgesia (32.4%), anticoagulants (16.2%), and steroids (16.2%) were the most commonly administered drugs. The mean length of hospitalization was 7.6±4.5 days, with only one patient (2.7%) requiring intensive care unit admission and assisted ventilation.

Conclusion

The overall prognosis was good since only one patient has passed away, while all others recovered and, subsequently, were discharged. Manifestations, laboratory investigations, and management modalities should be utilized promptly to enhance the prognosis and obtain better outcomes.

## Introduction

Since December 2019, many reports have been found in the literature reporting the new outbreak of the novel coronavirus (SARS-CoV-2), initially discovered in Wuhan, China. Coronavirus disease of 2019 (COVID-19), which is caused by SARS-CoV-2, started spreading across the world and was announced as a pandemic on March 11, 2020. Most COVID-19 symptomatic cases have mild to moderate presentations with a fever, unproductive cough, gastrointestinal symptoms, myalgia, and others [1]. Many approaches for the management of these cases have been innovated; however, the disease continues to affect many people worldwide. The virus causes an acute respiratory infection, primarily acute respiratory distress syndrome [2], and a vast array of pulmonary and extrapulmonary complications, mainly observed in patients with severe COVID-19 [3].

The extrapulmonary complications include septic shock, disseminated intravascular coagulation, myocardial injury, and acute kidney injury [3,4]. Moreover, it has been found that contracting the virus might exacerbate the severity of any underlying comorbidity [5-7]. Sickle cell disease (SCD) is a hereditary beta-hemoglobinopathy that produces abnormal hemoglobin, which causes the red blood cells (RBCs) to have an abnormal sickle shape. Patients with SCD suffer from multiple crises during their lifespan, most commonly the vaso-occlusive crises [8], which can cause immense pain and are the leading cause of hospitalization for SCD patients [9]. The second leading cause of hospitalization is acute chest syndrome, which results from the abnormal rheology of the RBCs where they form clusters and occlude the pulmonary microcirculation, leading to severe hypoxia and infarction. Furthermore, it is the leading cause of death in SCD. This process leads to an increase in cytokine release, which causes inflammation in the airways [8,10].

The prevalence of SCD within Saudi Arabia is relatively high, with an estimated 145/10,000 cases [11]. It was reported that one-fourth of admissions into Jazan hospitals was SCD, with the majority suffering from a vaso-occlusive crisis (VOC) followed by acute chest syndrome (ACS) [12]. Studies have shown that COVID-19 patients with SCD commonly present with fever, VOC, and ACS [13-16]. Nonetheless, COVID-19 can cause ACS and possibly trigger a VOC, as seen in multiple case reports [17-19]. There is a pressing demand for researching several aspects of COVID-19, a highly infectious disease, in order to better understand its effects on SCD patients in Saudi Arabia, also to achieve the best management protocol.

## Materials and methods

Study settings and population

This was a retrospective chart review study with data collected from a multi-center in Saudi Arabia between August 2020 and January 2021. We evaluated 33 patients with sickle cell anemia/disease who had confirmed diagnosis of COVID-19 by reverse transcription-polymerase chain reaction (RT-PCR). The diagnosis of COVID-19 was confirmed by using the polymerase chain reaction tests based on the nasopharyngeal swabs of the included patients. No specific criteria other than having concomitant COVID-19 infection and SCD were applied for our population to include all the relevant patients. Moreover, an institutional review board approval with reference number (H-02-J-002) was also obtained before conducting this study and data collection inauguration.

Data collection

The data of the current study were collected from the hospital information system and filled in our case report form. The form consisted of various sheets including the basic demographics of the study population, the present and past medical history and current medications, vital signs at baseline and during hospitalization, laboratory tests that were conducted for the patients, and the clinical outcomes that were observed in each patient during hospitalization, in addition to the management modalities that were approached with these patients. Moreover, if any patient had a previous history of hospitalization, these data were also included in this study. The severity of the conditions were also evaluated based on the characteristics of the patients’ conditions, using previously published criteria for this purpose. After data collection has finished, senior physicians reviewed and checked its clarity and usefulness for the analysis. Additionally, all of the underlying comorbidities were assessed and evaluated by experienced hematologists before establishing the appropriate diagnosis.

Statistical analysis

All analyses were done using Statistical Package for the Social Sciences (SPSS) v26 (IBM Statistics, Armonk, NY). Continuous variables were represented as means, and standard deviation and nominal variables were presented as counts and percentages. Regression models could not be reliably built due to the relatively small number of the included patients. The Spearman rank correlation coefficient (rho) was used to determine the relationship between different predictors and patients’ prognosis [20,21]. Correlation coefficient values of 0.10, 0.20, and 0.30 were interpreted as relatively small, typical, and relatively large [22], and the

## Results

Baseline characteristics

A total of 33 patients were included in the analysis, with a mean age of 10.75+9.11 years. The oldest patient included was 40 years old, while the youngest was two years old. Nearly, all patients were Saudi (n = 32; 96.9%) and 48.4% of them were females. Most of the patients (n = 24; 72.7%) did not suffer from any comorbidities, while six patients (18.1%) had asthma, two patients (6.1%) had glucose-6-phosphate dehydrogenase deficiency and one patient (3.1%) had methylmalonic acidemia plus seizures. Moreover, hydroxyurea was the only reported home medication in 15 patients (45.5%; Table 1).

**Table 1:** Baseline characteristics of the included patients (N=33) N: numbers, SD: standard deviation.

Variables		Count	%
Age; mean±SD		10.75+9.11	
Nationality	Saudi	32	96.9
	Non-Saudi	1	3.1
Gender	Female	16	48.4
	Male	17	51.6
Existing of comorbidity	None	24	72.7
	Asthma	6	18.1
	G6PD deficiency	2	6.1
	Methylmalonic acidemia	1	3.1
Home medication	Hydroxyurea	15	45.5
	None	18	54.5

**Figure 1:**
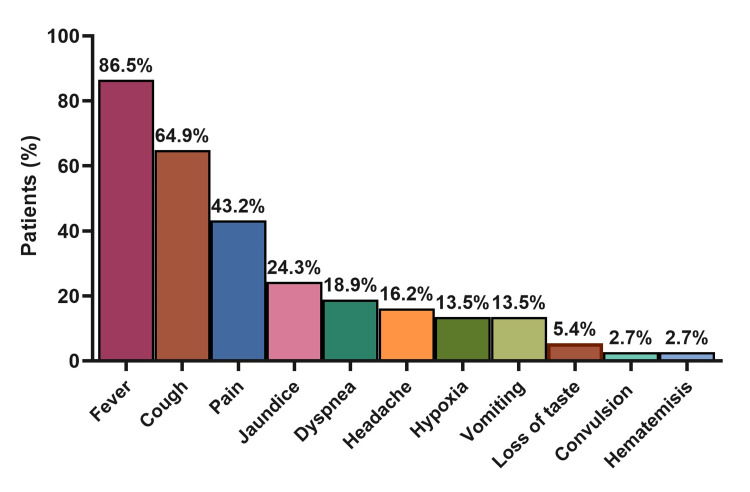
Summary of presenting symptoms for admitted patients (N=22) N: numbers.

**Figure 2:**
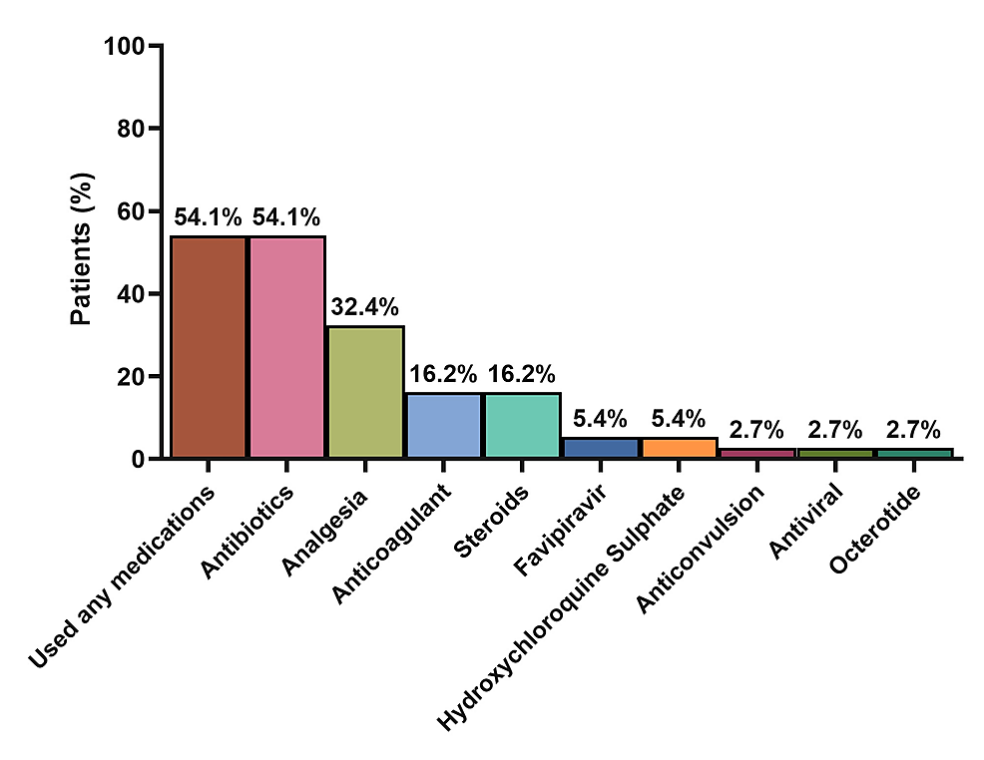
Summary of medication usage among admitted patients (N=22) N: numbers.

Twenty-two patients were admitted (59.5%) for different reasons as shown in Table 2. VOC only (n= 6; 27.3%), fever (n= 6; 27.3%), ACS (n= 5; 22.7%), and VOC combined with other conditions (n= 4; 18.2%) were the main causes for patients’ admission. Fever was the most prominent presenting symptom in admitted patients (86.5%), followed by cough (64.9%), pain (43.2%), jaundice (24.3%), dyspnea (18.9%), and headache (16.2%; Figure 1). During hospitalization, 54.1% of the patients received at least one medication, while antibiotics (54.1%), analgesia (32.4%), anticoagulants (16.2%), and steroids (16.2%) were the most commonly administered drugs (Figure 2).

**Table 2:** Different reasons for hospital admission (N=22) N: numbers.

Reason of admission	Count	%
Acute chest syndrome	5	22.7
Fever	6	27.3
Hemolytic crisis	1	4.5
Vaso‐occlusive crises	6	27.3
Vaso‐occlusive crises, convulsion	1	4.5
Vaso‐occlusive crises, acute chest syndrome	1	4.5
Vaso‐occlusive crises, hemolytic crisis	2	9.1

Lab parameters and chest X-ray findings

The summary of different lab parameters recorded for the admitted patients is presented in Table 3. For chest X-ray findings, 13 patients (35.1%) reported having a normal examination, bilateral ground-glass opacities (n=8; 21.6 %), unilateral infiltrations (n=1; 2.7%), and non-available data (n=15; 40.5%).

**Table 3:** Summary of different lab parameters for hospitalized patients (N=22) N: numbers.

Lab parameter	Mean	Standard deviation
White blood cells (10^9^/L)	15.0	7.0
Neutrophils (%)	7.8	5.7
Lymphocytes (%)	5.3	3.9
Hemoglobin (g/dL)	8.5	1.5
Platelets (× 10^3^/µL)	380.1	193.6
C-reactive protein (mg/L)	4.1	5.7
Erythrocyte sedimentation rate (mm/h)	32.0	23.1
Partial thromboplastin time (seconds)	38.4	16.8
Prothrombin time (seconds)	16.4	6.3
International normalized ratio	1.3	0.5
Total bilirubin (mg/dL)	2.7	1.7
Direct bilirubin (mg/dL)	0.8	0.7
Aspartate transaminase (IU/L)	91.7	156.0
Alanine transaminase (IU/L)	34.9	33.1
Lactate dehydrogenase (IU/L)	636.3	429.5
Alkaline phosphatase (IU/L)	278.2	436.6

Prognosis of hospitalized patients

The mean length of hospitalization was 7.6±4.5 days, with only one patient (2.7%) requiring intensive care unit (ICU) admission and assisted ventilation. The overall prognosis was good since only one case has passed away, at an age of 21 days only and with no administration of hydroxyurea, while all other cases recovered and, subsequently, were discharged (Table 4). The main highlight of correlation results is that medications affected the length of hospital stay. There was a relatively large negative correlation between hospital stay duration and having medications at home prior to admission (rho= −0.57; p-value=0.005) or during hospital admission (rho= −0.45; p-value=0.035). Besides, steroids and anti-coagulant have shown a relatively large positive correlation with ICU admission, assisted ventilation, and death; however, all of those outcomes were documented in one patient so the interpretation of these results should be made with caution. Noteworthy, age, gender, comorbidities, or the presence of fever on presentation did not show a significant effect on the tested outcomes (Table 5).

**Table 4:** Prognosis hospitalized patients (N=22) N: numbers, SD: standard deviation, ICU: intensive care unit.

Outcomes		Count	%
Length of hospitalization (days); mean±SD		7.6±4.5	
ICU admission	No	36	97.3
	Yes	1	2.7
Assisted ventilation	No	36	97.3
	Yes	1	2.7
Death	No	36	97.3
	Yes	1	2.7

**Table 5:** Correlation matrix of different predictors and patients’ prognosis *Statistically significant, ICU: intensive care unit.

Variables	Correlation results	ICU admission	Assisted ventilation	Length of hospitalization	Death
Age	Spearman's rho	0.2	0.2	0.05	0.2
	p-value	0.239	0.239	0.847	0.239
Gender	Spearman's rho	0.18	0.18	0.08	0.18
	p-value	0.284	0.284	0.724	0.284
Co-morbidities	Spearman's rho	0.09	0.09	−0.24	0.09
	p-value	0.608	0.608	0.292	0.608
Home medication	Spearman's rho	0.14	0.14	−0.57	0.14
	p-value	0.417	0.417	0.005*	0.417
Medications given at hospital	Spearman's rho	0.15	0.15	−0.45	0.15
	p-value	0.364	0.364	0.035*	0.364
Fever	Spearman's rho	0.07	0.07	0.17	0.07
	p-value	0.698	0.698	0.44	0.698
Steroids	Spearman's rho	0.38	0.38	0.15	0.38
	p-value	0.021*	0.021*	0.517	0.021*
Anti-coagulants	Spearman's rho	0.34	0.34	0.19	0.34
	p-value	0.039*	0.039*	0.405	0.039*

## Discussion

Acknowledging how COVID-19 impacts SCD patients can help guide their management and treatment protocols and provide the scientific field with further insight into this novel infectious disease. Therefore, our study aims to elaborate more on patients' clinical data and outcomes with concomitant COVID-19 and SCD, trying to understand the potential effect of COVID-19 on these patients. We found that among the 33 patients with COVID-19 and SCD, most of them, 70.3%, did not have any underlying comorbidities, while asthma was present in 16.2% of these patients.

The best way to understand these findings would be by comparing them to the results of the Surveillance Epidemiology of Coronavirus (COVID-19) Under Research Exclusion Overview (SECURE-SCD) registry, which was inaugurated by the Medical College of Wisconsin aiming at collecting and storing data about SCD patients who suffered from COVID-19 [14,23]. The registry was mainly designed to help identify all the potential features of this phenomenon and help healthcare practitioners establish the appropriate treatment and interventional approaches based on the most commonly reported features, with an easily accessible link [24]. Up to March 26, 2021, a total of 751 cases with SCD and COVID-19 were recorded, with a mean age of 22.07 (14.69). Among these patients, 52.73% were females, and the majority were not Hispanic/Latino. Regarding the type of the disease, Hb SS disease was present in 65.14% of the total population, followed by Hb SC (21.98%), and Hb S beta and thalassemia (6.62%) diseases. Regarding the presence of comorbidities, the registry shows that asthma together with cardiovascular diseases were the most common comorbidities among patients over 18 years of age, while asthma only was the most prevalent comorbidity among patients below this age, which is consistent with our findings. Moreover, pain that required hospitalization or emergency department admission followed by ACS were the most common symptoms that patients experienced within the last three years, according to the registry results.

Our results also showed that only one patient died (2.7%) among our study population. This is similar to the rate of deaths reported by the SECURE-SCD registry up to March 26, which was 2.53% in a population with a mean age of 22.07 years. The rate reported by the SECURE-SCD registry was even higher in the March-May 2020 cohort, with an estimated mortality rate of 7%, and an estimated ICU admission rate of 11%, with a mean age of <40 years [23]. Another French cohort study reported that the mortality rate was 2% only [14]. These rates are lower than the one reported by Minniti et al. [25], which estimated 10.6% among their population that had a median age of 34 years old. The case fatality rate of COVID-19 is hugely variable by country and differs according to many factors, and therefore, we could not compare these rates to the general case-fatality rates of COVID-19. However, evidence shows that the case fatality rate for COVID-19 alone is <1%, but further evidence per each country is still needed. Besides, we found that age, gender, comorbidities, or the presence of fever on presentation did not show a significant effect on the tested outcomes. COVID-19 induces generalized inflammation, which can cause serious multiple organ damage and complicate the infection [26,27]. Therefore, assessment of the laboratory parameters should be adequately done to avoid these complications.

The SECURE-SCD registry shows that most COVID-19 patients with SCD (55.5%) usually suffer from mild infections. Moreover, only 7.5% of the population required ICU admission, which is higher than our estimated rate (2.7%) for our population. On the other hand, the French cohort study reported a higher ICU admission rate (20%) among their population [14]. This might be attributable to the severity of the underlying medical condition and the compliance to the medical treatment. According to the SECURE-SCD registry, 52.6% of the patients administered hydroxyurea, while in our study, 40.5% of the population received the same medication. Hydroxyurea might be useful in reducing the inflammatory mediators, and it plays a major role in nitric oxide synthesis and regulation, which can enhance the microvasculature in the potential patients and enhance the prognosis of the disease [28-30]. Additionally, Heparin and Azithromycin were the most commonly prescribed medications during hospitalization in the SECURE-SCD registry, while in our population, antibiotics and analgesics were the most commonly prescribed drugs.

Additionally, the SECURE-SCD registry showed emergency department visits, hospitalization, and transfusions were the most prevalent interventions for their population. Therefore, the difference in these characteristics might be the cause of the different reported characteristics between our findings and those reported by the registry. These findings imply that early identification of the symptoms and reasons of admissions of SCD patients with COVID-19 is essential for the early intervention against developing a severe status that may end up with death. Therefore, clinicians should care for the presence of VOC, fever, or ACS, in addition to the laboratory tests and the administration of adequate management modalities to obtain better outcomes in these patients.

Our findings were limited by the small size, which has restricted us from performing further innovative analytical correlations that may have furtherly elaborated more on the characteristics of COVID-19 in SCD patients. Besides, data were retrospectively affected which has limited our findings as some parameters were missing, like interval time from the initial signs and symptoms and the admission to the hospital, and the specific therapeutic measures that were taken for each type of SCD. We suggest that further research be inaugurated to understand the infection in these patients and achieve better prognostic outcomes. Our study did not differentiate the type of SCD, whether it is sickle cell anemia or sickle cell with Hb, which might affect the prognosis of the disease.

## Conclusions

Our findings indicate that VOC, fever, and ACS are the most common reasons for hospital admissions in SCD patients with COVID-19. Asthma was the most prevalent comorbidity, and hydroxyurea was the most commonly administered home medication. Almost all patients recovered, while only one patient required ICU admission and died. Clinicians should care for the presenting manifestations, laboratory investigations, and management modalities to enhance the prognosis and obtain better outcomes.
